# MicroRNA-7 Inhibits Multiple Oncogenic Pathways to Suppress HER2Δ16 Mediated Breast Tumorigenesis and Reverse Trastuzumab Resistance

**DOI:** 10.1371/journal.pone.0114419

**Published:** 2014-12-22

**Authors:** Felicia C. Huynh, Frank E. Jones

**Affiliations:** 1 Department of Cell and Molecular Biology, Tulane University, New Orleans, Louisiana, United States of America; 2 Department of Cell and Molecular Biology, Tulane Cancer Center, Tulane University, New Orleans, Louisiana, United States of America; University of South Alabama, United States of America

## Abstract

The oncogenic isoform of HER2, HER2Δ16, is expressed with HER2 in nearly 50% of HER2 positive breast tumors where HER2Δ16 drives metastasis and resistance to multiple therapeutic interventions including tamoxifen and trastuzumab. In recent years microRNAs have been shown to influence multiple aspects of tumorigenesis and tumor cell response to therapy. Accordingly, the HER2Δ16 oncogene alters microRNA expression to promote endocrine resistance. With the goal of identifying microRNA suppressors of HER2Δ16 oncogenic activity we investigated the contribution of altered microRNA expression to HER2Δ16 mediated tumorigenesis and trastuzumab resistance. Using a gene array strategy comparing microRNA expression profiles of MCF-7 to MCF-7/HER2Δ16 cells, we found that expression of HER2Δ16 significantly altered expression of 16 microRNAs by 2-fold or more including a 4.8 fold suppression of the miR-7 tumor suppressor. Reestablished expression of miR-7 in the MCF-7/HER2Δ16 cell line caused a G1 cell cycle arrest and reduced both colony formation and cell migration activity to levels of parental MCF-7 cells. Suppression of miR-7 in the MCF-7 cell line resulted in enhanced colony formation activity but not cell migration, indicating that miR-7 suppression is sufficient to drive tumor cell proliferation but not migration. MiR-7 inhibited MCF-7/HER2Δ16 cell migration through a mechanism involving suppression of the miR-7 target gene EGFR. In contrast, miR-7 inhibition of MCF-7/HER2Δ16 cell proliferation involved a pathway where miR-7 expression resulted in the inactivation of Src kinase independent of suppressed EGFR expression. Also independent of EGFR suppression, reestablished miR-7 expression sensitized refractory MCF-7/HER2Δ16 cells to trastuzumab. Our results demonstrate that reestablished miR-7 expression abolishes HER2Δ16 induced cell proliferation and migration while sensitizing HER2Δ16 expressing cells to trastuzumab therapy. We propose that miR-7 regulated pathways, including EGFR and Src kinase, represent targets for the therapeutic intervention of refractory and metastatic HER2Δ16 driven breast cancer.

## Introduction

Breast cancer is the most commonly diagnosed cancer in North American women and the second leading cause of cancer related deaths. At least five different molecular breast cancer subtypes have been identified and each subtype is associated with significantly different patient outcomes [1], [2]. The HER2 positive subtype represents 20–30% of breast cancers and patients with HER2 positive tumors have the shortest overall survival. Furthermore, patients with tumor expression of an activated and presumably highly oncogenic HER2 receptor have an even worse prognosis [3].

One tumor specific event that results in clinical activation of HER2 is expression of the alternatively spliced and constitutively active HER2Δ16 isoform. HER2Δ16 is co-expressed with HER2 in nearly 50% of HER2 positive breast tumors [4]. Significantly, 90% of patients with tumor expression of HER2Δ16 present with disseminated metastatic disease. In contrast, breast tumors that overexpress wild-type HER2, but lack detectable HER2Δ16 expression, are significantly associated with favorable clinicopathological markers including lymph node negative cancer [4]. When overexpressed in breast tumor cells, HER2Δ16 promotes resistance to multiple endocrine therapies [5], [6], as well as, the HER2 targeted therapy trastuzumab [4]. These clinical and experimental observations suggest that HER2Δ16 expression drives HER2 positive breast cancer to an aggressive and therapeutic refractory metastatic disease. Although the molecular basis of HER2Δ16 oncogenic activity remains to be deciphered, recent studies indicate that HER2Δ16 expression alters microRNA (miR) expression to evade therapeutic intervention [5], [6].

MiRs are a class of short non-coding single-stranded RNAs that regulate gene expression. Specific binding of miRs to the 3′ untranslated region (UTR) of target gene mRNA results in suppressed target gene translation which may also be associated with degradation of the target gene mRNA. Although miRs play key roles during normal developmental processes, deregulation of miR expression has been noted in several human cancers where miRs have been shown to have both oncogenic and tumor suppressor functions [7]–[9]. MiR-7 has been shown to suppress breast tumorigenesis by reducing expression of multiple target genes including epidermal growth factor receptor (EGFR) [10], p21-activated kinase 1 (PAK1) [11], focal adhesion kinase (FAK) [12], and krupple-like factor 4 (KLF4) [13]. Here we show that breast tumor cells expressing oncogenic HER2Δ16 have reduced expression of the miR-7 tumor suppressor. Accordingly, reintroduced miR-7 expression suppressed HER2Δ16 oncogenic activity by inhibiting expression of EGFR and independently inactivating Src kinase.

## Materials and Methods

### Cell lines

MCF-7 cells were purchased from American Type Culture Collection and cultured according to their instructions. The stable MCF-7 cell line expressing pcDNA3 or the two independent cell lines expressing pcDNA3-HER2Δ16 and referred to here as MCF-7/pcDNA, MCF-7/HER2Δ16H, and MCF-7/HER2Δ16M1, respectively, have been described elsewhere [14]. For stable suppression of EGFR to generate the pooled MCF-7/HER2Δ16/EGFRKD cell line, MCF-7/HER2Δ16H cells were transfected with the MISSION shRNA plasmid-DNA TRCN0000121329 targeting EGFR (Sigma) or a pLKO.1 (Sigma) negative control using Fugene6 (Roche). For stable suppression of miR-7 to generate the pooled MCF-7/miR-7KD cell line, MCF-7 cells were transfected with the miRZip-7 anti-miR-7 microRNA construct MZIP7-PA-1 (System Biosciences) or the pGreenPuro Scramble Hairpin Control construct MZIP000-PA-1 (System Bioscience) using Fugene6. For stable expression of miR-7 to generate the pooled MCF-7/HER2Δ16H/miR-7 cell line, MCF-7/HER2Δ16H cells were transfected with the miR-7 expression vector MI0000263 (Origene) using the NEON Transfection System exactly as described by the manufacturer (Invitrogen). At two days post-transfection all pooled cell lines were selected for two days in 1 µg/ml puromycin (Gibco) and then maintained at 0.2 µg/ml puromycin.

### Micro RNA expression profiling

Four independent total RNA samples from MCF-7/pcDNA and MCF-7/HER2Δ16H cells were purified using the miRVANA RNA Isolation System (Life Technologies) according to the manufacturer's instructions and RNA integrity was confirmed using a Bioanalyzer (Agilent). Microarray assay was performed and analyzed using a service provider (LC Sciences) and LC-Science microRNA array miRHuman_11.0 which detects miR transcripts listed in Sanger miRBase Release 11.0. The microRNA array data presented in this publication have been deposited in NCBI's Gene Expression Omnibus [15] and are accessible through GEO Series accession number GSE62848 (http://www.ncbi.nlm.nih.gov/geo/query/acc.cgi?acc=GSE62848).

### Quantification of miR-7 expression

Triplicate total RNA samples were purified using the miRVANA RNA Isolation System (Life Technologies) according to the manufacturer's instructions and RNA integrity was confirmed using a Bioanalyzer (Agilent). First-strand complementary DNA (cDNA) was synthesized from 1.0 µg of total RNA in a 20 µl reaction volume using the Superscript III First-Strand Synthesis System (Life Technologies) with a RNU44 (Applied Biosystems) or dme-miR-7 RT-primer (Applied Biosystems) exactly as described by the manufacturer. Following reverse transcription, 180 µl of water was added to the cDNA reaction and 2 µl of the diluted cDNA was used in a 20 µl TaqMan MicroRNA Assay using the RNU44 or dme-miR-7 probe set with TaqMan Universal PCR Master Mix (Applied Biosystems) in the 7500 Fast Real-Time PCR system (conditions as follows: 40 cycles of 50°C for 2 min, 95°C for 10 min, and 60°C for 15 sec followed by 60°C for 1 min), exactly as described by the manufacturer (Applied Biosystems). The CT analysis for each reaction was performed using the supplied 7500 Software v2.0.5 (Applied Biosystems) and miR-7 levels were normalized to RNU44 and relative expression was calculated using the 2^-ΔΔCT^ method.

### Western blot analysis

Total cell lysates were prepared in RIPA Buffer (10 mM NaPO_4_, pH 7.2, 150 mM NaCl, 1 mM EDTA, 0.1% SDS, 1% Na-deoxycholate, 1% Nonidet P40) containing Complete EDTA-free Protease Inhibitor Cocktail (Roche) and PhosSTOP phosphatase inhibitor (Roche). Lysates were solubilized in NuPAGE LDS Sample Buffer (4X) (Life Technologies) with NuPAGE Sample Reducing Agent (10X) (Life Technologies) added to 1X. Lysates were separated by electrophoresis with 20 µg of protein per lane in a NuPAGE 4–12% Bis-Tris Gel (Life Technologies) and transferred to an Immobilon-FL 0.45 µm Pore Size Transfer Membrane (Millipore) using a Trans-Blot Semi-Dry Electrophoretic Transfer Cell (Bio-Rad). Membranes were blocked and all antibody dilutions were performed in 5% BSA (Sigma) in TBST (10 mM Tris, pH 7.5, 150 mM NaCl, 0.1% Tween-20). All washes were performed in TBST. Primary antibodies included α-tubulin #05829 (Upstate Biotechnology), HER2 #MS-325 (Neomarkers), EGFR #4267 (Cell Signaling Technology), PAK1 #2602 (Cell Signaling Technology), Src #2110 (Cell Signaling Technology), Phospho-Src Y416 #6943 (Cell Signaling Technology), FAK #3285 (Cell Signaling Technology), and FAK Y576/577 #3281 (Cell Signaling Technology). Secondary antibodies were Alexa Fluor Conjugated Affinity Purified Anti-Rabbit or Anti-Mouse IgG (Life Technologies) detected using an Odyssey Infrared Imaging System (LiCor Biosciences).

### Colony formation assay

Cells were plated at 1,000 cells per well in a 6-well plate and media was replaced every two days for 12 days total. For trastuzumab treatments, trastuzumab was added to media at 10 µg/ml and media was replaced with fresh control or trastuzumab containing media every two days during the experiment. Colonies were fixed in 100% methanol and stained with crystal violet. Colony number and diameter were calculated using a ColCount Colony Counter (Oxford Optronix) and data was analyzed using the supplied statistical software.

### Cell cycle analysis

Cells were synchronized in serum-free MEM for 24 hrs and then cultured in MEM with 10% FBS for an additional 24 hrs. Trypsin treated cells were fixed in 100% ethanol overnight. Fixed cells were stained with Guava Cell Cycle Reagent (Millipore) and cell cycle was analyzed in a Guava Easy Cyte Mini Base System (Millipore) using the supplied statistical software exactly as described previously [16].

### Cell migration assay

Cell migration was determined using the xCELLigence System (Roche) with the CIM-Plate 16 and RTCA DP Instrument (Roche) according to the manufacturer's instructions. Briefly, 40,000 cells were added to the upper CIM-Plate 16 chamber in media containing 0.2% fetal bovine serum (FBS). Media with 10% FBS was added to the lower CIM-Plate 16 chamber and the CIM-Plate 16 was incubated in the RTCA DP Instrument for 48 hrs. Cell migration as a function of real-time changes in electrical impedance was monitored by the xCELLigence System. Cell Index (referred to here as Migration Index) and standard deviation of replicates were calculated using the supplied RTCA Software (Roche).

## Results and Discussion

### HER2Δ16 alters expression of multiple miRs involved in breast tumorigenesis

**Figure 1:**
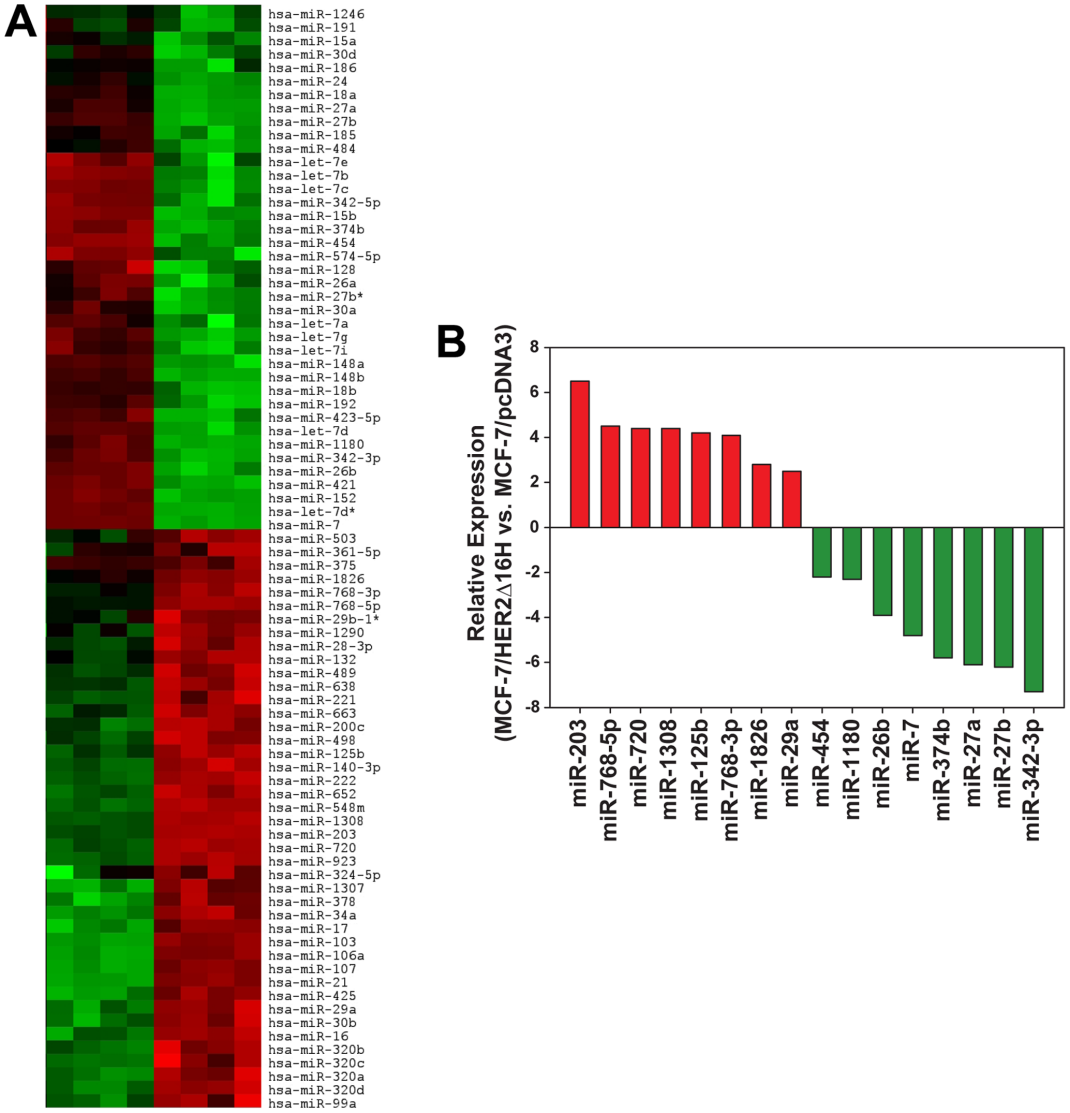
Altered miR expression in response to HER2Δ16 expression in MCF-7 cells. (A) Heat map of all miRs significantly (*p*<0.01) altered by 1.2-fold or greater or (B) by 2-fold or greater in MCF-7/HER2Δ16H cells when compared to MCF-7/pcDNA cells. Data represents four independent RNA samples for each cell line.

Clinical and experimental evidence suggests that wild-type HER2 is a relatively weak breast oncogene when compared to the aggressive therapeutic refractory phenotype associated with breast tumors expressing the constitutively active HER2Δ16 isoform [4]–[6], [17]. MiRs potentially regulate multiple properties of tumorigenesis and therapeutic response. Accordingly, we have recently shown that HER2Δ16 expression alters expression of miR-15a/16 and miR-342 to promote endocrine resistance of breast tumor cells [5], [6]. To investigate the potential role of miR expression contributing to HER2Δ16 driven tumorigenesis we compared global miR expression profiles between parental MCF-7 breast cancer cells (MCF-7/pcDNA) and a MCF-7 cell line with ectopic expression of HER2Δ16 (MCF-7/HER2Δ16H) [4]. Probing a LC Sciences miR array containing 837 unique human miRs we found that HER2Δ16 expression significantly (*p*<0.01) altered the expression of 82 miRs (Fig. 1A) with 16 miRs altered by 2-fold or greater (Fig. 1B). Of the 16 miRs altered in HER2Δ16 expressing cells at least three are consistent with a role in HER2Δ16 driven tumorigenesis. For example, we have previously shown that the suppression of miR-342-3p (the most dramatically altered miR) contributes to endocrine resistance of HER2Δ16 expressing breast tumor cells [6]. Upregulation of miR-125b expression has been suggested to regulate chemosensitivity of breast tumor cells [18], [19]. For the current studies we focused our attention on the 4.8 fold suppression of miR-7. Significantly, miR-7 is considered a potent tumor suppressor miR and has been shown to regulate expression of multiple target genes in breast tumor cells [10]–[13].

Although the exact mechanism mediating HER2Δ16 suppression of miR-7 remains to be established our previously published data suggests that the Jumonji/ARID1 B (JARID1B) transcriptional repressor may play a role. JARID1B is a breast oncogene most dramatically overexpressed in HER2 positive breast tumors [20]–[22]. We have shown that JARID1B transcriptionally represses the expression of multiple tumor suppressor miRs in breast tumor cell lines [16]. Significantly, over 90% of the JARID1B regulated miRs, including miR-7, are also similarly altered by HER2Δ16 expression. Although experimental validation is required, these observations raise the possibility that JARID1B transcriptionally represses multiple miRs, including miR-7, in HER2Δ16 expressing breast tumor cells.

### MiR-7 suppresses breast tumor cell proliferation and HER2Δ16 driven cell migration

We have previously shown that HER2Δ16 expression significantly potentiates MCF-7 cell proliferation, migration/invasion, xenograft tumor formation, and resistance to multiple therapeutic interventions; whereas, expression of wild-type HER2 failed to enhance a single tumorigenic property of MCF-7 cells [4]–[6], [17]. Given the potent oncogenic activity of HER2Δ16 and the clinical association of HER2Δ16 with metastatic breast cancer we investigated the impact of miR-7 activity on HER2Δ16 driven tumorigenesis.

**Figure 2:**
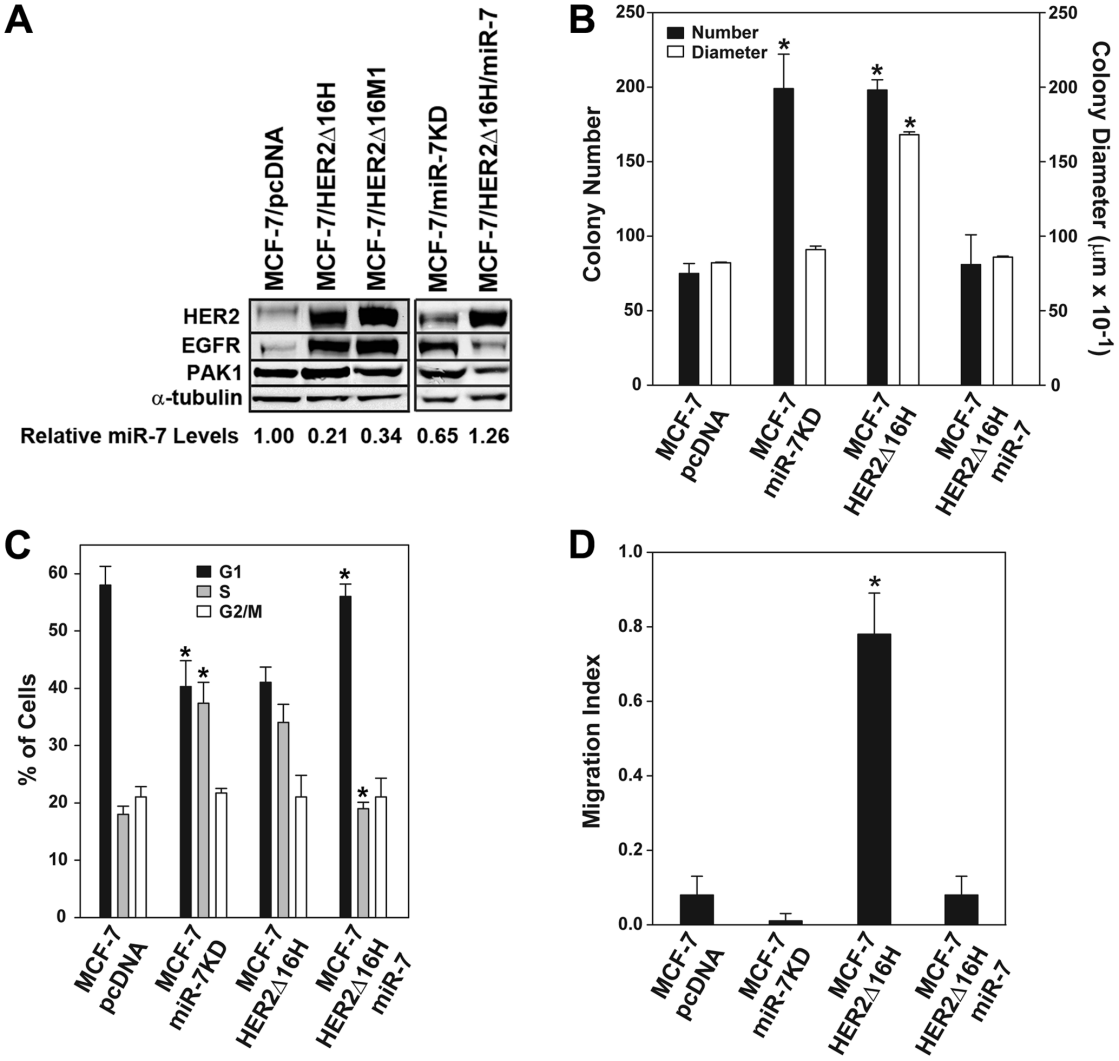
Altered miR-7 expression regulates breast tumor cell proliferation and migration. Mir-7 expression was stably suppressed in the MCF-7 cell line to generate the MCF-7/miR-7KD cell line and a miR-7 expression plasmid was stably introduced into the MCF-7/HER2Δ16H cell line to generate the MCF-7/HER2Δ16H/miR-7 cell line. (A) Western blot analysis of the MCF-7 vector control, MCF-7/pcDNA and the HER2Δ16 expressing MCF-7/HER2Δ16H and MCF-7/HER2Δ16M1 and cell lines with altered miR-7 expression. Relative miR-7 expression levels are indicated below each lane as determined by qRT-PCR. (B) Colony formation assay where colony number and diameter were calculated using a ColCount Colony Counter with supplied statistical software. Asterisk indicates cell lines significantly greater than MCF-7/pcDNA and MCF-7/HER2Δ16/miR-7 as determined by paired Student's *t* test (*p*<0.006). (C) Cell cycle analysis was performed in a Guava Easy Cyte Mini Base System with supplied statistical software. Asterisk indicates cell cycle phase significantly different from parental cell line as determined by paired Student's *t* test (*p*<0.01). (D) Cell migration was determined in an xCELLigence CIM-Plate 16 with the RTCA DP Instrument for 48 hrs. Cell Index (referred to here as Migration Index) was calculated using the supplied RTCA Software. Asterisk indicates that MCF-7/HER2Δ16H is significantly greater than the other tested cell lines as determined by paired Student's *t* test (*p*<0.005). (B–D) The data represents the mean +/− SE of at least three independent experiments.

MiR-7 expression was suppressed by 3 to 5-fold in two independent HER2Δ16 expressing MCF-7 cell lines when compared to the MCF-7 parental cell line (Fig. 2A). Modulation of miR-7 expression had a dramatic impact on the miR-7 target gene EGFR [23] with the highest levels of EGFR expression associated with reduced levels of miR-7 in the MCF-7/HER2Δ16H, and MCF-7/HER2Δ16M1 cell lines (Fig. 2A). Consistent with these observations, knockdown of miR-7 expression in the MCF-7 cell line, MCF-7/miR-7KD, resulted in enhanced EGFR expression whereas reintroduced miR-7 expression in the MCF-7/HER2Δ16H cell line, MCF-7/HER2Δ16H/miR-7, resulted in suppressed EGFR expression. A similar but less dramatic impact on the miR-7 target gene PAK1 [11] was also observed in the modified MCF-7 cell lines. Altered miR-7 and EGFR expression was continuously monitored and remained stable in the MCF-7/miR-7KD and MCF-7/HER2Δ16H/miR-7 cell lines for greater than 20 cell passages. These results suggest that selective suppression of miR-7 and subsequent restoration of EGFR expression occurs in response to ectopic HER2Δ16 expression in MCF-7 cells.

We next determined the impact of altered miR-7 expression levels on breast tumor growth in a colony formation assay. Consistent with previous reports [4] expression of HER2Δ16 in the MCF-7 cell line had a significant impact on colony number and diameter (Fig. 2B). In concordance with its role as a tumor suppressor, reestablished miR-7 expression to generate the MCF-7/HER2Δ16H/miR-7 cell line significantly reduced the number and diameter of MCF-7/HER2Δ16H colonies to levels equivalent to the MCF-7/pcDNA cell line (Fig. 2B). Conversely, suppression of MCF-7 miR-7 expression to generate the MCF-7/miR-7KD cell line resulted in a significant increase in colony number to levels equivalent to ectopic expression of HER2Δ16 (Fig. 2B). We investigated the mechanistic basis of miR-7 tumor suppressor activity by performing cell cycle analysis. We observed a significant increase in cells arrested at G1 of the cell cycle with lower levels of S phase cells in the MCF-7/pcDNA and MCF-7/HER2Δ16/miR-7 cell lines both with enhanced expression of miR-7 (Fig. 2C). In contrast, G1 arrest is released in the MCF-7/miR-7KD and MCF-7/HER2Δ16 cell lines with suppressed miR-7 expression and these cells exhibit an increase in S phase cells (Fig. 2C). Cell cycle analysis suggests that miR-7 suppresses tumor cell growth by inducing a G1 arrest with a concomitant reduction in proliferating S phase cells. This result corroborates a recent study that described a similar G1 arrest when miR-7 was expressed in Chinese hamster ovary (CHO) cells [24]. Taken together our results indicate that suppression of miR-7 is both necessary and sufficient to promote cell cycle progression and significantly enhance colony formation of breast tumor cells.

We have previously shown that ectopic expression of HER2Δ16 induces a dramatic migration/invasion phenotype in the non-invasive MCF-7 cell line [4]. To determine if altered miR-7 expression regulates MCF-7 cell migration we performed an xCELLigence migration assay on cell lines with altered miR-7 expression. Our results indicate that suppression of miR-7 expression in the MCF-7/miR-7KD cell line is not sufficient to promote MCF-7 cell migration (Fig. 2D). Consistent with our previous observations HER2Δ16 expression in the MCF-7/HER2Δ16H cell line caused a significant increase in MCF-7 cell migration (Fig. 2D). Interestingly, reestablished expression of miR-7 in the MCF-7/HER2Δ16H/miR-7 cell line completely abolished cell migration and reduced the migration index to levels observed for parental MCF-7 cells (Fig. 2D). These results indicate that suppression of miR-7 is necessary but, in contrast to tumor cell proliferation, not sufficient to promote breast tumor cell migration.

### MiR-7 regulates multiple oncogenic pathways that influence HER2Δ16 driven cell proliferation and migration

**Figure 3:**
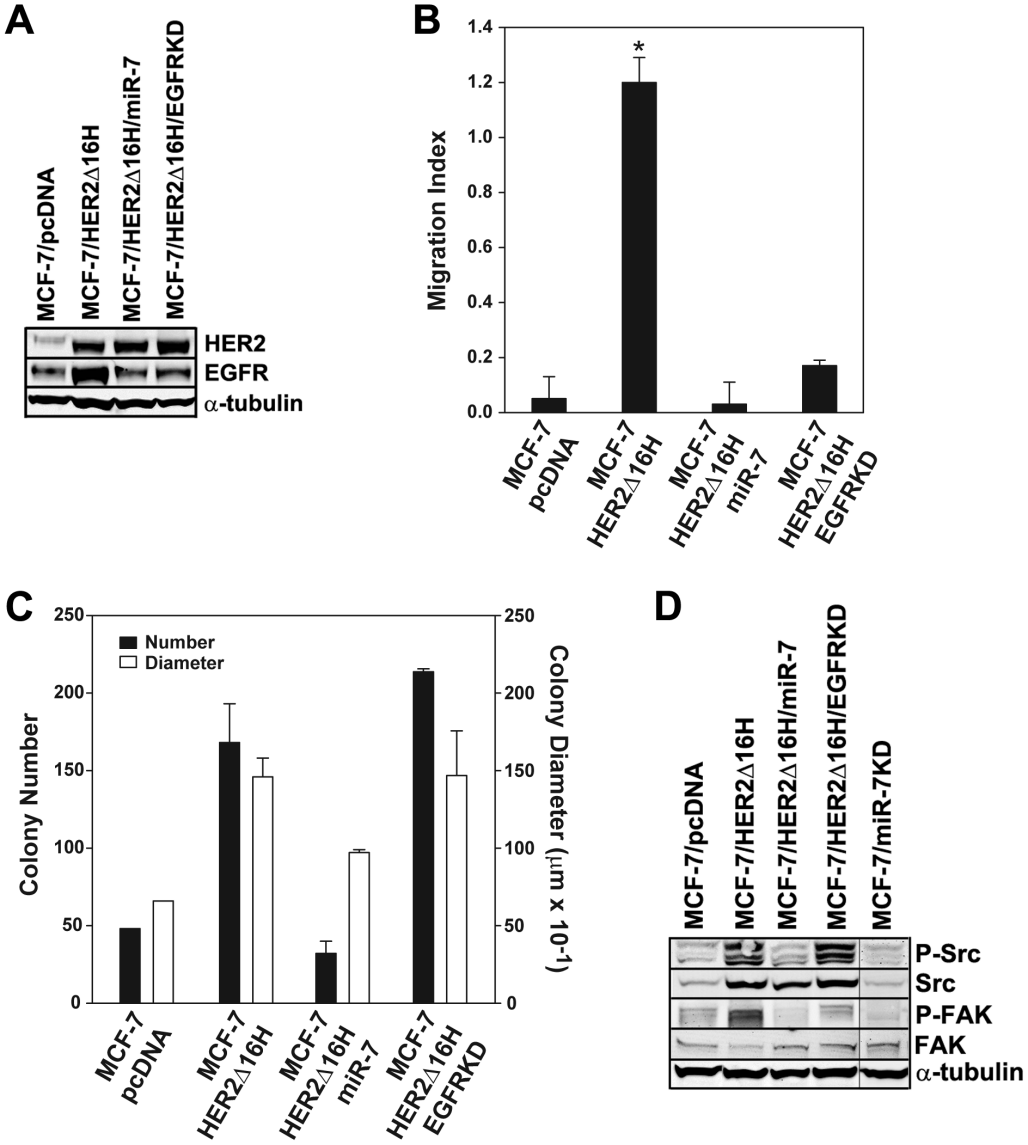
MiR-7 regulates HER2Δ16 induced cell migration and proliferation through different oncogenic pathways. A miR-7 expression plasmid was stably introduced into the MCF-7/HER2Δ16H cell line to generate the MCF-7/HER2Δ16H/miR-7 cell line and EGFR was stably knocked down by shRNA to generate the MCF-7/HER2Δ16H/EGFRKD cell line. (A) Altered expression of EGFR in the indicated cell lines was confirmed by western blot analysis. (B) Cell migration was determined in an xCELLigence CIM-Plate 16 with the RTCA DP Instrument for 48 hrs. Cell Index (referred to here as Migration Index) was calculated using the supplied RTCA Software. Asterisk indicates that MCF-7/HER2Δ16H is significantly greater than the other tested cell lines as determined by paired Student's *t* test (*p*<0.001). (C) Colony formation assay where colony number and diameter were calculated using a ColCount Colony Counter with supplied statistical software. Differences between MCF-7/HER2Δ16H and MCF-7/HER2Δ16H/EGFRKD were insignificant as determined by paired Student's *t* test. (D) Western blot analysis of the indicated cell lines probed for Src kinase (Src), Src kinase activated phosphorylated at Y416 (P-Src), and Src kinase activity through phosphorylation of FAK at Y576/577 (P-FAK). (B,C) The data represents the mean +/− SE of at least three independent experiments.

We investigated the impact of altered miR-7 expression on multiple reported gene targets including FAK [12], insulin-like growth factor 1 receptor (IGF1R) [25], PAK1 [11], and EGFR [10], however, in our experimental system, EGFR was the only target that was consistently altered in response to modulated miR-7 expression (Fig. 2A, Fig. 3D, and data not shown). We therefore determined if EGFR is the miR-7 target gene that contributes to HER2Δ16 oncogenic activity. Using shRNA we inhibited EGFR expression in the MCF-7/HER2Δ16H cell line (MCF-7/HER2Δ16H/EGFRKD) (Fig. 3A) and determined the impact of suppressed EGFR expression on HER2Δ16 mediated colony formation and migration. Suppression of EGFR expression in the MCF-7/HER2Δ16H/EGFRKD cell line essentially abolished cell migration of MCF-7/HER2Δ16H cells reducing their migration activity to levels similar to parental MCF-7/pcDNA and MCF-7/HER2Δ16H/miR-7 cells (Fig. 3B). Surprisingly, suppression of EGFR failed to impact colony formation activity in the MCF-7/HER2Δ16H/EGFRKD cell line (Fig. 3C). EGFR therefore appears to be an essential component of the HER2Δ16 cell migration pathway; however, EGFR signaling is dispensable for HER2Δ16 colony formation activity. These results indicate that although miR-7 regulation of EGFR expression significantly impacts cell migration, a different miR-7 regulated pathway(s) influences MCF-7/HER2Δ16H colony formation activity.

We have previously shown that Src kinase is an important effector of multiple HER2Δ16 oncogenic activities including cell migration/invasion and colony formation [4]. Accordingly, RNAi suppression of Src kinase expression or dasatinib inhibition of Src kinase activity results in the complete loss of HER2Δ16 oncogenic activity in multiple biological assays [4]. We therefore examined the influence of miR-7 on Src kinase expression and activity. A slight decrease in Src protein was observed in the miR-7 expressing cell line MCF-7/HER2Δ16H/miR-7 (Fig. 3D). Src kinase is not a predicted direct target of miR-7 (www.targetscan.org) suggesting that the decrease in expression is due to indirect effects of miR-7 activity.

Interestingly, although miR-7 fails to directly target Src kinase, Src activation through phosphorylation of the regulatory Y416 was completely abolished in the MCF-7/HER2Δ16H/miR-7 cell line (Fig. 3D). Src activation was however retained in the MCF-7/HER2Δ16H/EGFRKD cell line (Fig. 3D) indicating that the loss of Src activation in the MCF-7/HER2Δ16H/miR-7 cell line was not due to miR-7 suppression of EGFR. Likewise, Src activation and expression levels remained relatively low in the MCF-7/miR-7KD cell line indicating that expression of EGFR is not sufficient to activate Src kinase (Fig. 3D).

We examined the impact of miR-7 on Src kinase activity. An important target of Src kinase activity is phosphorylation of FAK at Y576/577 [26] and as predicted this phosphorylation site is enhanced in the MCF-7/HER2Δ16H cell line (Fig. 3D; P-FAK) indicating that Src is active in this cell line. Loss of Src activation in the MCF-7/HER2Δ16H/miR-7 cell line also resulted in abolished Src activity as demonstrated by the loss of FAK Y576/577 phosphorylation (Fig. 3D). Loss of EGFR resulted in intermediate levels of FAK Y576/577 (Fig. 3D) indicating that EGFR is required for maximum Src activity and FAK phosphorylation in the MCF-7/HER2Δ16 cell line. The reduced levels of FAK phosphorylation may explain the loss of migration associated with EGFR suppression in the MCF-7/HER2Δ16H/EGFRKD cell line.

The mechanistic basis of miR-7 inactivation of Src kinase remains unclear. Common miR target prediction software (MiRanda, PicTar, and TargetScan) failed to detect a consensus miR-7 binding site in the *SRC* gene; however, imperfect miR-7 binding sites identified using RNAhybrid [27] were predicted to be located in the 3′-UTR and 5′-UTR of *SRC*. Although we detected robust suppression of the miR-7 target sequence using the MIR-REPORT (Applied Biosystems) reporter system, miR-7 failed to regulate the imperfect *SRC* miR-7 binding sites (data not shown) in the same experiment. The lack of direct Src kinase regulation by miR-7, suggests that miR-7 indirectly inactivates Src kinase in HER2Δ16 expressing cells through a novel miR-7 target gene or pathway. Src is activated through the actions of multiple different receptor tyrosine kinases, integrins, as well as, G-protein coupled receptors [26], [28]. It is possible that miR-7 suppresses Src activation by targeting one of these Src regulating pathways.

Taken together our results suggest that miR-7 inhibits HER2Δ16 induced cell migration through multiple pathways including suppression of EGFR expression and loss of Src kinase activity. MiR-7 inhibition of HER2Δ16 mediated cellular proliferation, on the other hand, was independent of EGFR suppression but likely involves a miR-7 regulated pathway that drives Src inactivation.

### MiR-7 sensitizes refractory HER2Δ16 expressing cells to trastuzumab

**Figure 4:**
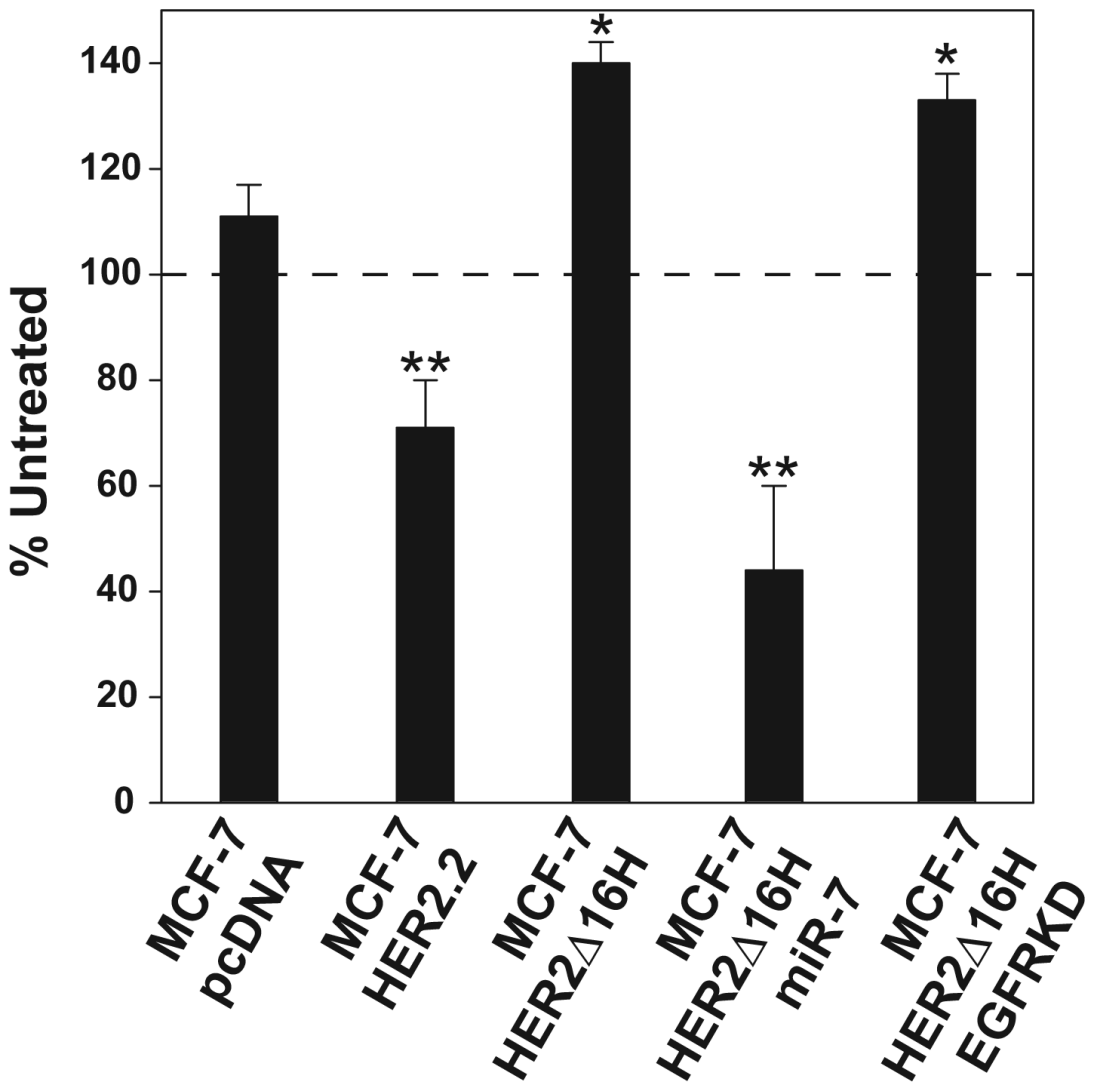
MiR-7 reverses trastuzumab resistance of HER2Δ16 expressing cells. Colony formation of each indicated cell line calculated using a ColCount Colony Counter with supplied statistical software. The MCF-7/HER2.2 cell line expresses wild-type HER2 and is included as a positive control. The data represents the percentage difference in colony number of trastuzumab treated compared to the untreated control +/− SE of at least three independent experiments. Single asterisk or double asterisks indicate trastuzumab treated cell lines with significantly enhanced (*p*<0.04) or reduced (*p*<0.02) colony formation, respectively. Significant differences were determined by paired Student's *t* test.

We have previously demonstrated that ectopic expression of HER2Δ16 in the MCF-7 cell line promotes trastuzumab resistance [4]. In fact we consistently observe enhanced growth of HER2Δ16 expressing cells in response to trastuzumab, implicating trastuzumab as a HER2Δ16 agonist [4]. We therefore determined if trastuzumab resistance of HER2Δ16 expressing cells is influenced by altered expression of miR-7 or EGFR. Consistent with our previously published results [4], trastuzumab significantly suppressed colony formation of MCF-7/HER2.2 cells expressing wild-type HER2; whereas trastuzumab significantly enhanced colony formation of the HER2Δ16 expressing MCF-7/HER2Δ16H cell line (Fig. 4). Suppression of EGFR expression failed to influence the response of MCF-7/HER2Δ16H/EGFRKD cells to trastuzumab as these cells also exhibited significantly enhanced colony formation activity in response to trastuzumab (Fig. 4). In contrast, the MCF-7/HER2Δ16H/miR-7 cell line with reestablished expression of miR-7 responded to trastuzumab treatment with a significant reduction in colony formation (Fig. 4). Importantly, miR-7 not only functions as a potent suppressor of HER2Δ16 tumorigenesis but also reverses HER2Δ16 induced trastuzumab resistance.

## Conclusions

**Figure 5:**
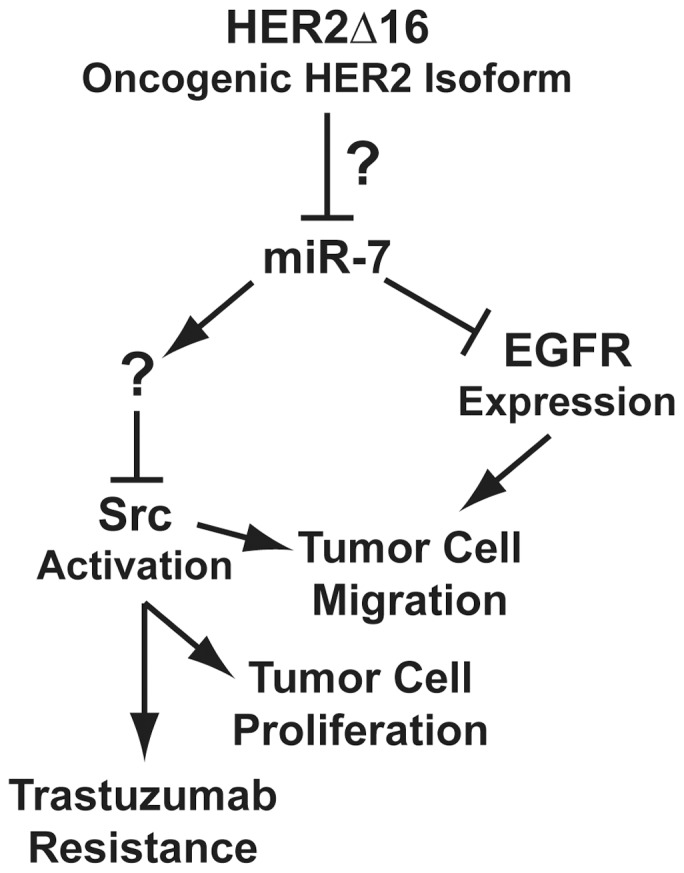
The influence of miR-7 regulated pathways on HER2Δ16 oncogenic activity. Expression of the HER2 oncogenic isoform, HER2Δ16, in the MCF-7 breast tumor cell line results in reduced expression of the miR-7 tumor suppressor. Reintroduced miR-7 expression results in direct repression of EGFR protein expression and indirect suppression of Src activation through a novel intermediate pathway(s). EGFR expression is required for HER2Δ16 driven cell migration, whereas activated Src is an obligate effector of multiple HER2Δ16 activities including trastuzumab resistance. We propose that reactivation of miR-7 expression would represent an efficacious therapeutic strategy to suppress HER2Δ16 driven metastatic disease and reverse trastuzumab resistance.

Despite the clinical use of the HER2 targeted therapy, trastuzumab, patients with HER2 positive breast tumors have the lowest disease specific survival and a significant percentage of HER2 positive patients fail to benefit from trastuzumab therapy [29], [30]. We have shown that 90% of patients with tumor expression of the HER2 isoform, HER2Δ16, also present with metastatic disease [4]. Furthermore, breast tumor cell expression of HER2Δ16 promotes trastuzumab resistance. We contend that successful treatment of HER2 positive metastatic breast cancer requires a strategy to disengage HER2Δ16 oncogenic signaling. To this end we show that HER2Δ16 suppresses expression of the miR-7 tumor suppressor and reestablished miR-7 expression significantly inhibits HER2Δ16 mediated tumor cell proliferation and migration and miR-7 sensitizes HER2Δ16 expressing cells to trastuzumab treatment (Fig. 5).

Although tumor delivery of miRs remains a significant clinical challenge, deciphering miR regulated pathways may identify suitable targets for therapy. Our findings that miR-7 suppression of HER2Δ16 oncogenic activity is mediated through inactivation of Src kinase and suppression of EGFR expression (Fig. 5) implies that targeting these pathways would also suppress HER2Δ16 tumorigenesis. Consistent with a potential role for EGFR in HER2Δ16 tumorigenesis, coexpression of EGFR in breast tumors with activated HER2 is associated with significantly shorter patient survival than patients with tumor expression of activated HER2 or EGFR alone [31]. In addition, we and others have demonstrated that targeting Src kinase sensitizes trastuzumab resistant tumors [4], [32] independent of EGFR expression. Moreover, we have shown that the Src kinase inhibitor dasatinib is a potent inhibitor of HER2Δ16 mediated breast tumorigenesis [4]. In conclusion, our current results showing miR-7 inactivation of Src kinase further implicates Src kinase as an obligate effector of trastuzumab resistance and HER2Δ16 oncogenic activity (Fig. 5).

## References

[1] Perou CM, Sorlie T, Eisen MB, van de Rijn M, Jeffrey SS, et al (2000) Molecular portraits of human breast tumours. Nature 406:747–752. 10963602 10.1038/35021093

[2] Sorlie T, Perou CM, Tibshirani R, Aas T, Geisler S, et al (2001) Gene expression patterns of breast carcinomas distinguish tumor subclasses with clinical implications. Proc Natl Acad Sci U S A 98:10869–10874. 11553815 10.1073/pnas.191367098 PMC58566

[3] Thor AD, Liu S, Edgerton S, II DM, Kasowitz KM, et al (2000) Activation (tyrosine phosphorylation) of ErbB-2 (HER-2/neu): A study of incidence and correlation with outcome in breast cancer. J Clin Oncol 18:3230–3239. 10986055 10.1200/JCO.2000.18.18.3230

[4] Mitra D, Brumlik MJ, Okamgba SU, Zhu Y, Duplessis TT, et al (2009) An oncogenic isoform of HER2 promotes metastatic breast cancer and trastuzumab resistance. Mol Cancer Ther 8:2152–2162. 19671734 10.1158/1535-7163.MCT-09-0295

[5] Cittelly DM, Das PM, Salvo VA, Fonseca JP, Burow ME, et al (2010) Oncogenic HER2Δ16 suppresses miR-15a/16 and deregulates BCL-2 to promote endocrine resistance of breast tumors. Carcinogenesis 31:2049–2057. 20876285 10.1093/carcin/bgq192 PMC2994280

[6] Cittelly DM, Spoelstra NS, Edgerton SM, Richer JK, Thor AD, et al (2010) Suppression of miR-342 expression contributes to anti-estrogen resistance in breast tumor cells. Mol Cancer 9:317. 21172025 10.1186/1476-4598-9-317 PMC3024251

[7] Esquela-Kerscher A, Slack FJ (2006) Oncomirs - microRNAs with a role in cancer. Nat Rev Cancer 6:259–269. 16557279 10.1038/nrc1840

[8] Pencheva N, Tavazoie SF (2013) Control of metastatic progression by microRNA regulatory networks. Nat Cell Biol 15:546–554. 23728460 10.1038/ncb2769 PMC4678324

[9] Verghese ET, Hanby AM, Speirs V, Hughes TA (2008) Small is beautiful: microRNAs and breast cancer-where are we now? J Pathol 215:214–221. 18446835 10.1002/path.2359

[10] Webster RJ, Giles KM, Price KJ, Zhang PM, Mattick JS, et al (2009) Regulation of epidermal growth factor receptor signaling in human cancer cells by microRNA-7. J Biol Chem 284:5731–5741. 19073608 10.1074/jbc.M804280200

[11] Reddy SD, Ohshiro K, Rayala SK, Kumar R (2008) MicroRNA-7, a homeobox D10 target, inhibits p21-activated kinase 1 and regulates its functions. Cancer Res 68:8195–8200. 18922890 10.1158/0008-5472.CAN-08-2103 PMC3636563

[12] Kong X, Li G, Yuan Y, He Y, Wu X, et al (2012) MicroRNA-7 inhibits epithelial-to-mesenchymal transition and metastasis of breast cancer cells via targeting FAK expression. PLoS ONE 7:e41523. 22876288 10.1371/journal.pone.0041523 PMC3410899

[13] Okuda H, Xing F, Pandey PR, Sharma S, Watabe M, et al (2013) miR-7 suppresses brain metastasis of breast cancer stem-like cells by modulating KLF4. Cancer Res 73:1434–1444. 23384942 10.1158/0008-5472.CAN-12-2037 PMC3576138

[14] Naresh A, Thor AD, Edgerton SM, Torkko KC, Kumar R, et al (2008) The HER4/4ICD estrogen receptor coactivator and BH3-only protein is an effector of tamoxifen-induced apoptosis. Cancer Res 68:6387–6395. 18676864 10.1158/0008-5472.CAN-08-0538 PMC2538429

[15] Edgar R, Domrachev M, Lash AE (2002) Gene Expression Omnibus: NCBI gene expression and hybridization array data repository. Nucleic Acids Res 30:207–210. 11752295 10.1093/nar/30.1.207 PMC99122

[16] Mitra D, Das PM, Jones FE (2010) JUMONJI/ARID1 B (JARID1B) promotes breast tumor cell cycle progression through epigenetic repression of micro RNA let-7e. J Biol Chem 286:40531–40535. 10.1074/jbc.M111.304865 PMC3220509 21969366

[17] Sridhar J, Sfondouris ME, Bratton MR, Nguyen TL, Townley I, et al (2014) Identification of quinones as HER2 inhibitors for the treatment of trastuzumab resistant breast cancer. Bioorg Med Chem Lett 24:126–131. 24355130 10.1016/j.bmcl.2013.11.064

[18] Wang H, Tan G, Dong L, Cheng L, Li K, et al (2012) Circulating MiR-125b as a marker predicting chemoresistance in breast cancer. PLoS ONE 7:e34210. 22523546 10.1371/journal.pone.0034210 PMC3327688

[19] Zhou M, Liu Z, Zhao Y, Ding Y, Liu H, et al (2010) MicroRNA-125b confers the resistance of breast cancer cells to paclitaxel through suppression of pro-apoptotic Bcl-2 antagonist killer 1 (Bak1) expression. J Biol Chem 285:21496–21507. 20460378 10.1074/jbc.M109.083337 PMC2898411

[20] Barrett A, Madsen B, Copier J, Lu PJ, Cooper L, et al (2002) PLU-1 nuclear protein, which is upregulated in breast cancer, shows restricted expression in normal human adult tissues: a new cancer/testis antigen? Int J Cancer 101:581–588. 12237901 10.1002/ijc.10644

[21] Barrett A, Santangelo S, Tan K, Catchpole S, Roberts K, et al (2007) Breast cancer associated transcriptional repressor PLU-1/JARID1B interacts directly with histone deacetylases. Int J Cancer 121:265–275. 17373667 10.1002/ijc.22673

[22] Yamamoto S, Wu Z, Russnes HG, Takagi S, Peluffo G, et al (2014) JARID1B is a luminal lineage-driving oncogene in breast cancer. Cancer Cell 25:762–777. 24937458 10.1016/j.ccr.2014.04.024 PMC4079039

[23] Kefas B, Godlewski J, Comeau L, Li Y, Abounader R, et al (2008) microRNA-7 inhibits the epidermal growth factor receptor and the Akt pathway and is down-regulated in glioblastoma. Cancer Res 68:3566–3572. 18483236 10.1158/0008-5472.CAN-07-6639

[24] Sanchez N, Gallagher M, Lao N, Gallagher C, Clarke C, et al (2013) MiR-7 triggers cell cycle arrest at the G1/S transition by targeting multiple genes including Skp2 and Psme3. PLoS ONE 8:e65671. 23762407 10.1371/journal.pone.0065671 PMC3675065

[25] Jiang L, Liu X, Chen Z, Jin Y, Heidbreder CE, et al (2010) MicroRNA-7 targets IGF1R (insulin-like growth factor 1 receptor) in tongue squamous cell carcinoma cells. Biochem J 432:199–205. 20819078 10.1042/BJ20100859 PMC3130335

[26] Ishizawar R, Parsons SJ (2004) c-Src and cooperating partners in human cancer. Cancer Cell 6:209–214. 15380511 10.1016/j.ccr.2004.09.001

[27] Rehmsmeier M, Steffen P, Hochsmann M, Giegerich R (2004) Fast and effective prediction of microRNA/target duplexes. RNA 10:1507–1517. 15383676 10.1261/rna.5248604 PMC1370637

[28] Playford MP, Schaller MD (2004) The interplay between Src and integrins in normal and tumor biology. Oncogene 23:7928–7946. 15489911 10.1038/sj.onc.1208080

[29] Arteaga CL, Sliwkowski MX, Osborne CK, Perez EA, Puglisi F, et al (2012) Treatment of HER2-positive breast cancer: current status and future perspectives. Nat Rev Clin Oncol 9:16–32. 10.1038/nrclinonc.2011.177 22124364

[30] Slamon DJ, Leyland-Jones B, Shak S, Fuchs H, Paton V, et al (2001) Use of chemotherapy plus a monoclonal antibody against HER2 for metastatic breast cancer that overexpresses HER2. N Engl J Med 344:783–792. 11248153 10.1056/NEJM200103153441101

[31] DiGiovanna MP, Stern DF, Edgerton SM, Whalen SG, Moore D 2nd, et al (2005) Relationship of epidermal growth factor receptor expression to ErbB-2 signaling activity and prognosis in breast cancer patients. J Clin Oncol 23:1152–1160. 15718311 10.1200/JCO.2005.09.055

[32] Zhang S, Huang WC, Li P, Guo H, Poh SB, et al (2011) Combating trastuzumab resistance by targeting SRC, a common node downstream of multiple resistance pathways. Nat Med 17:461–469. 21399647 10.1038/nm.2309 PMC3877934

